# CD1c-Expression by Monocytes – Implications for the Use of Commercial CD1c^+^ Dendritic Cell Isolation Kits

**DOI:** 10.1371/journal.pone.0157387

**Published:** 2016-06-16

**Authors:** Martine Schrøder, Guro Reinholt Melum, Ole J. B. Landsverk, Anna Bujko, Sheraz Yaqub, Einar Gran, Henrik Aamodt, Espen S. Bækkevold, Frode L. Jahnsen, Lisa Richter

**Affiliations:** 1 Department of Pathology, Oslo University Hospital, Oslo, Norway; 2 Centre for Immune Regulation, University of Oslo, Oslo, Norway; 3 Department of Gastrointestinal Surgery, Oslo University Hospital, Oslo, Norway; 4 Department of Otolaryngology, Lovisenberg Diakonale Hospital, Oslo, Norway; 5 Department of Cardiothoracic Surgery, Oslo University Hospital, Oslo, Norway; 6 Tumor Immunology Group, Department of Pathology, Oslo University Hospital, Oslo, Norway; University of Bergen, NORWAY

## Abstract

Conventional dendritic cells (cDCs) comprise a heterogeneous population of cells that are important regulators of immunity and homeostasis. CD1c^+^ cDCs are present in human blood and tissues, and found to efficiently activate naïve CD4^+^ T cells. While CD1c is thought to specifically identify this subset of human cDCs, we show here that also classical and intermediate monocytes express CD1c. Accordingly, the commercial CD1c (BDCA-1)^+^ Dendritic Cell Isolation Kit isolates two distinct cell populations from blood: CD1c^+^CD14^−^ cDCs and CD1c^+^CD14^+^ monocytes. CD1c^+^ cDCs and CD1c^+^ monocytes exhibited strikingly different properties, including their differential regulation of surface marker expression, their levels of cytokine production, and their ability to stimulate naïve CD4^+^ T cells. These results demonstrate that a commercial CD1c (BDCA-1)^+^ Dendritic Cell Isolation Kit isolates two functionally different cell populations, which has important implications for the interpretation of previously generated data using this kit to characterize CD1c^+^ cDCs.

## Introduction

The cells of the mononuclear phagocyte system are subdivided into dendritic cells (DCs), monocytes and macrophages. Among these, DCs are the most adept at promoting naïve T cell activation. Plasmacytoid and conventional (c) DCs are specialized subsets identified by expression of distinct surface markers [1]. cDCs comprise two subsets: Blood CD1c^+^ DCs are SIRPα^+^ and assigned to the cDC2 lineage, as opposed to SIRPα^-^CD141^+^DNGR1^+^ cDCs that constitute the cDC1 lineage [2]. CD1c^+^ DCs are the major subset of DCs among human peripheral blood mononuclear cells (PBMCs) [1,3] and in a variety of human tissues [4–8]. CD1c^+^ DCs have been found to efficiently induce naïve CD4^+^ T cells [3,4]. The antigen CD1c (BDCA-1) is a member of the CD1 family of transmembrane glycoproteins which are structurally related to major histocompatibility complex (MHC) proteins. CD1 proteins mediate presentation of lipid and glycolipid antigens, including mycobacterial cell wall components, to T cells [9]. cDCs constitute only a small fraction of blood and tissue cells, rendering enrichment and isolation techniques favorable. CD1c^+^ DCs were early on defined as negative for lineage (lin) markers (CD3, CD16, CD19, CD20, CD56) and for CD141 (BDCA-3), as well as the plasmacytoid DC markers CD303 (BDCA2) and CD304 (BDCA-4), with a minor proportion expressing CD14 [1]. This definition is presently used as basis for the application of a commercially available CD1c (BDCA-1)^+^ Dendritic Cell Isolation Kit (Miltenyi Biotec), suggesting that after the removal of CD19^+^ B cells, only CD1c^+^ cDCs are positively selected from the remaining cell suspension using CD1c-targeting antibody [10]. However, here we report that CD1c is expressed by a fraction of human monocytes in blood and mucosal tissues, and demonstrate that this widely-used kit isolates CD1c^+^ cell populations that show striking phenotypical and functional differences.

## Materials and Methods

### Samples

Blood samples were obtained from healthy adult blood donors at Department of Immunology and Transfusion Medicine, Oslo University Hospital, Oslo, Norway. Citrate phosphate dextrose solution was used as anticoagulant. Peripheral blood mononuclear cells (PBMCs) were isolated by Lymphoprep™ (Stemcell technologies, Vancouver, Canada) density gradient centrifugation.

Mucosal tissue samples from the distal duodenum / proximal jejunum were obtained during Whipple procedure on pancreatic cancer patients, distant from the tumor. The tissue was immediately transported to our laboratory and prepared for immunohistochemistry (see below) and for flow cytometry. In the latter situation epithelial cells were removed by three washing steps with 2mM EDTA in PBS for 20min at 37°C. The remaining lamina propria was digested in RPMI containing 0.25mg/ml liberase (Roche, Mannheim, Germany) and 20U/ml DNase I (Roche) at 37°C. Mononuclear cells were enriched by Lymphoprep™ (Stemcell technologies) density gradient centrifugation.

Samples of nasal mucosa were obtained from the lower edge of the inferior turbinate during surgery for septum deviation on healthy donors (n = 7), or donors with grass or birch allergy out of season (n = 4). The tissue was finely minced and digested as above.

Samples of bronchial mucosa were obtained from patients with non-small cell lung cancer (n = 5). The lung lobe was removed and mucosa was scraped off from inside the largest bronchus distant to the tumor region. The tissue was finely minced and digested as above.

All participants provided written consent, and the study was approved by the Norwegian Regional Committee for Medical Research Ethics (Oslo, Norway).

### Flow Cytometry and antibodies

CD19-PE-Cy7 (HIB19), CD14-APC-Cy7 (HCD14), CD45-v510 (HI30), HLA-DR-bv605 (L243), CD1c-PerCP, -PE, -APC (all L161), SIRPα-PE-Cy7 (SE5A5), CD1a-PE (HI149), CD16-PE (3G8), DNGR1-PE (8F9), CD115-PE (9-4D2-1EA), CCR2-bv421 (MCP1), CD11b-bv421 (ICRF44) and TREM1-PE (TREM-26) were from Biolegend (San Diego, CA). CD103-FITC, -PE and –APC (all B-Ly7) were from eBioscience (San Diego, CA). CD3-APC (sk7), CD20-PE (L27), CD56-PE (B159), CD11c-APC, –PE (both s-HCL-3) and CD11c-v450 (B-Ly6) were from BD Bioscience (San Jose, CD). Calprotectin-PE (MAC387; targeting subunit S100A9) was from AbD Serotec (Oxfordshire, United Kingdom). PD-L1-APC (PDL1.3.1) and CD207 (DCGM4) were from Beckman Coulter (Fullerton, CA). For staining procedures of intracellular proteins, cells were permeabilized using the FoxP3 / transcription factor staining buffer set according to the manufacturer’s instructions (eBioscience). Samples were acquired on a BD LSR Fortessa and data analyzed with FlowJo 10.0.1 software (Tree Star, Eugene, OR). Compensation was performed using single-stained OneComp eBeads (eBioscience). Dead cells were stained with propidium iodide, To-PRO-1 (Molecular Probes, Life Technologies, Carlsbad, CA), fixable Viability Dye eFluor450 or eFluor780 (eBioscience) and excluded during analysis. Cell aggregates (identified on FSC-A versus FSC-H and SSC-A versus SSC-W scatter plots) were excluded during analysis. Shaded areas in plots represent corresponding FMO or matched isotype controls.

### Isolation, culture and sorting

CD1c^+^ DCs were purified with magnetic cell sorting (MACS) using anti-CD1c (BDCA-1) micro beads according to the manufacturer’s instructions [10] (median purity 97%; Miltenyi Biotec, Bergisch Gladbach, Germany). In brief, CD19^+^ cells were depleted using anti-CD19-coated magnetic beads, and CD1c^+^ cells were isolated using biotinylated anti-CD1c and anti-biotin-beads. Isolated cells were cultured for 48h in RPMI containing 10% heat-inactivated fetal calf serum, 1% penicillin-streptomycin and L-glutamine. Where indicated 40ng/ml human recombinant TGFβ (R&D Systems, Minneapolis, MN) was added. For cytokine expression analysis, blood cells were presorted using the CD1c-isolation kit. For analysis of TNF expression cultured cells were then stimulated for 4.5h with 1μg/ml LPS (Sigma, St. Louis, MO) with addition of 10μg/ml Brefeldin A (Sigma) for 3.5h. For analysis of IL-10 expression cultured cells were stimulated for 18h with 1μg/ml LPS and 0.7μg/ml Golgi-Stop (BD Bioscience). For analysis of IL-12p40 expression cultured cells were pre-treated with 100ng/ml INFγ (R&D Systems) for 1h, then stimulated for 24h with 1μg/ml LPS (Sigma, St. Louis, MO) and 10μg/ml Brefeldin A (Sigma). For mixed lymphocyte reaction experiments, blood cells were presorted using the CD1c-isolation kit (Miltenyi Biotec) and cultured for 48h. CD45^+^HLA-DR^+^CD11c^+^CD14^-^ DCs and CD45^+^HLA-DR^+^CD11c^+^CD14^+^ monocytes were then sorted on a BD ARIAIIu (BD). To this end we used the antibodies CD45-APC-Cy7 (H130), HLA-DR-PerCP-Cy5.5 (L243), CD14-PacificBlue (HCD14), SIRPα-PE-Cy7 (SE545) (all Biolegend) and CD11c-APC (S-HCL-3) (BD Bioscience). For generation of an enrichment protocol without the use of FACS-sorting, pre-depletion of monocytes was performed using CD14-MACS micro beads (Miltenyi Biotec) following the manufacturer’s protocol.

### Immunohistochemistry

Biopsies from small intestinal mucosa were mounted on a slice of carrot, using OCT compound (Tissue-Tek; Miles Laboratories, Elkhart, IN), then embedded in OCT compound, snap-frozen in liquid nitrogen, and stored at −p°C. 4 μm cryosections were cut and air-dried overnight at RT, post-fixed in acetone for 10 min, wrapped in aluminum foil, and stored at −20°C until used. Cryosections were incubated for 1 hour with anti-CD14 (clone 18D11, mIgG1, 1/500; Gift from Terje Espevik) and anti-CD1c (clone AD5-8E7, mIgG2, 1/10; Miltenyi Biotec), followed by Cy3-conjugated goat anti-mIgG1 (1/1500; Southern Biotech, Birmingham, AL) and alexa fluor 488-conjugated goat anti-mIgG2 (1/1000; Molecular Probes, Life Technologies) for 30 min. Sections incubated with the secondary antibodies alone were used as controls. Laser confocal microscopy was performed on an Olympus FV1000 (BX61WI) system (Olympus Corporation, Tokyo, Japan) with 60xLUMFI objective and 488nm, 543 and 633nm laser lines. Microscopy images were assembled in Photoshop CS4 and Illustrator CS6 (Adobe, San Jose, CA).

### Mixed lymphocyte reaction

Naïve CD4^+^ T cells were isolated using the MACS Naïve CD4^+^ T cell isolation kit (miltenyi biotech), and stained with CFSE (Life Technologies) according to the manufacturer’s instructions. APC subsets were pre-sorted on CD1c and FACS-sorted as described above, and equal numbers of cells (4,000–24,500) from each subset were cultured with naïve CFSE-stained CD4^+^ T cells at a ratio of 1:5 for 5–6 days in RPMI containing 10% heat-inactivated fetal calf serum, 1% penicillin-streptomycin and L-glutamine. As a control, equal amounts of T cells were cultured alone. For intracellular staining of cytokines, the co-cultures were stimulated with 25ng/ml PMA and 250ng/ml ionomycin for 3.5 h with addition of 10μg/ml brefeldin A. For CD4^+^ T cell phenotyping after co-culture experiments CD3–PE-Cy7 (UCH-T1), CD25-APC (2A4), T-bet-PE (o4-46) (all BD Bioscience), TNFα-bv605 (Mab11) and IL-10-bv421 (JES3-9D7) (both Biolegend) or matched isotype controls were used. For staining procedures of intracellular and nuclear proteins, cells were permeabilized using the FoxP3 / transcription factor staining buffer set according to the manufacturer’s instructions (eBioscience).

### Immunoassay

Supernatants from co-cultures were stored at -80°C until used. Cytokines were quantified using the Bio-Plex ProTM Human Treg Cytokine Panel (#171AL003M, Bio-Rad Laboratories, Hercules, CA) according to the manufacturers’ instructions.

### Statistics

Statistical analyses were performed using GraphPad Prism software (version 4; GraphPad Software, La Jolla, CA). A *P* value of less than 0.05 was considered significant. The tests used and magnitudes for *P* are indicated in each figure legend.

## Results

### Blood and mucosal tissue monocytes express CD1c

Numerous publications have used CD1c as a marker to isolate cDC2 cells from blood using a commercial bead-based isolation kit [11–19]. However, applying this kit we found that a substantial fraction of the positively sorted cells expressed high levels of CD14. This finding prompted us to investigate whether cDC2 cells purified using this kit were contaminated by monocytes.

**Fig 1:**
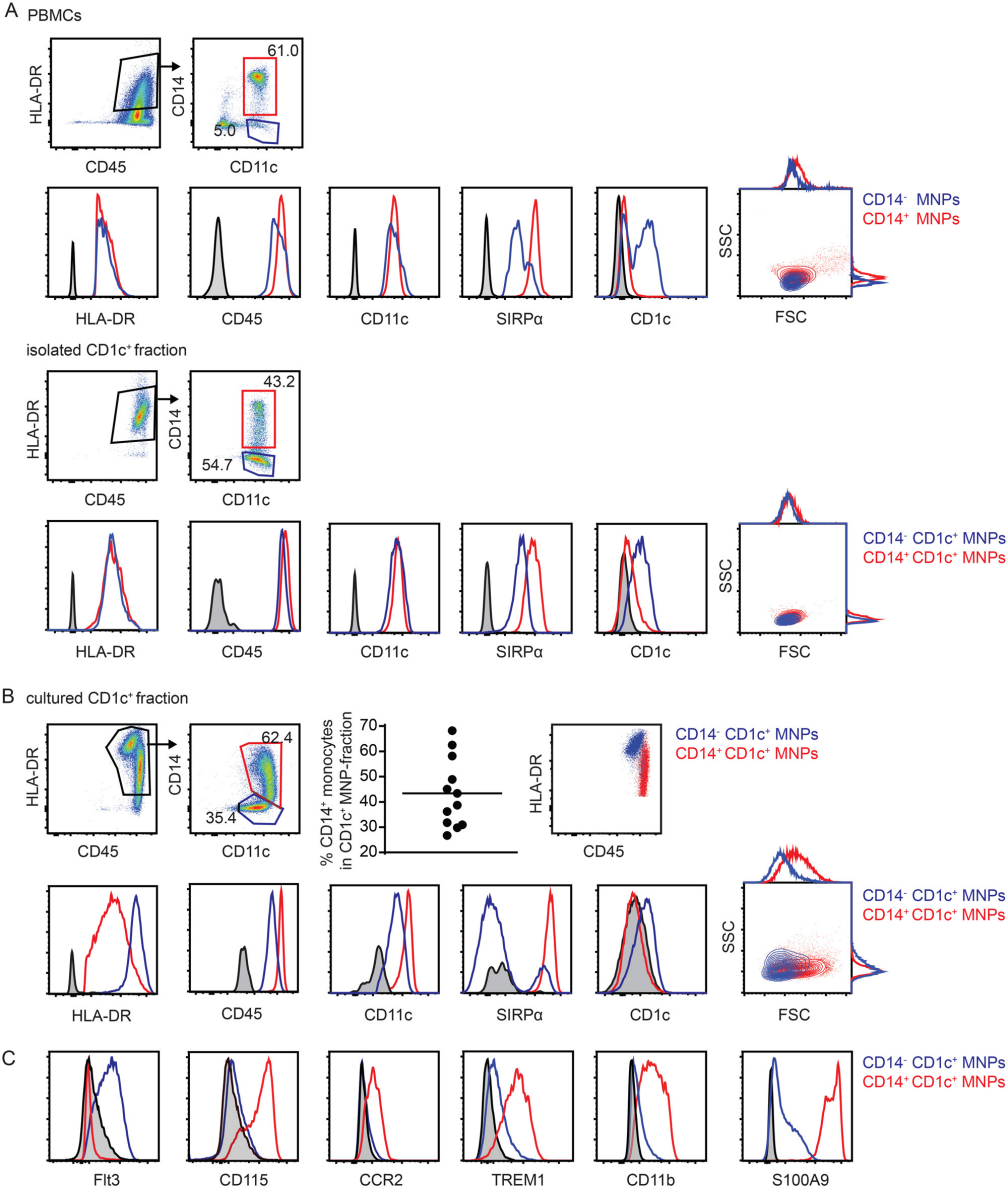
CD1c^+^ DC isolation kit isolates CD1c^+^ DCs and CD1c^+^ monocytes. (A) Flow cytometric phenotyping of the initial CD14^-^ and CD14^+^ populations in PBMCs (upper panel) and the resulting CD1c^+^CD14^-^ and CD1c^+^CD14^+^ populations after application of the CD1c^+^ DC isolation kit (lower panel). Cells are pre-gated on single, live cells (see S1 Fig). Data are representative for 6 donors. (B) Flow cytometric phenotyping of the CD1c^+^CD14^-^ and CD1c^+^CD14^+^ populations after 48h culture (phenotyping representative for 3 donors), and relative abundance of CD14^+^ cells among CD1c^+^ mononuclear phagocytes (n = 12). Cells are pre-gated on single, live cells. (C) Expression pattern of DC marker Flt3 as well as monocyte markers CD115, CCR2, TREM1, CD11b and S100A9 in CD14^-^ CD1c^+^ and CD14^+^CD1c^+^ populations. Data are representative for 2–3 donors. Cells are pre-gated on single, live CD45^+^HLA-DR^+^CD11c^+^ cells (see S1 Fig).

Using the CD1c (BDCA-1)^+^ Dendritic Cell Isolation Kit (Miltenyi Biotec) on PBMCs from healthy blood donors we found an enrichment of CD11c^+^CD14^-^ DCs among the CD45^+^HLA-DR^+^ mononuclear phagocytes in the CD1c^+^ fraction (Fig 1A, and see S1 Fig for full gating strategy). However, we simultaneously observed a considerable number of CD14^+^ cells in the isolated population (Fig 1A). In fact, flow cytometric analysis of the isolated fraction revealed a CD14^+^CD1c^+^ subset of mononuclear phagocytes which exhibited higher levels of SIRPα expression than CD14^-^CD1c^+^ mononuclear phagocytes, but comparable expression levels of markers used in the upstream gating strategy (Fig 1A).

Potential contaminating cells other than CD14^+^CD1c^+^ APCs were analyzed using the lineage markers CD3 (T lymphocytes), CD19 (B lymphocytes), CD20 (B lymphocytes) and CD56 (NK cells), and granulocyte marker CD15. The contribution of non-APCs to the CD1c^+^ fraction was consistently lower than 5% and mostly comprised CD3^+^ cells (S1 Fig).

Subsequent culture of isolated CD1c^+^ MNPs resulted in two populations of cells exhibiting pronounced differences in HLA-DR and CD14 levels, with HLA-DR^hi^ cells being CD14^-^ and HLA-DR^low^ cells being CD14^+^ (Fig 1B). Importantly, we observed a high inter-individual variation in abundance of CD14^+^ cells, ranging from 25 to 70% CD14^+^ cells among CD1c^+^ mononuclear phagocytes (mean 43.36 ± 13.68; n = 12) (Fig 1B). The fraction of SIRPα^+^ cells was decreased in the CD14^-^ population and expression of CD11c and CD45 was slightly down-regulated, whereas CD14^+^ cells increased in size in comparison to CD14^-^ cells, and down-regulated HLA-DR expression (Fig 1B). Expression of CD1c was lower in both populations after culture (Fig 1B). Growth factor receptor Fms-like tyrosine kinase 3 (Flt3) ligand-dependent development and therefore expression of Flt3 substantiates the identity of pre-cDC-derived cells [20–22], contrasting the M-CSF-dependent development of monocyte-derived cells [23,24]. Importantly, among the cultured cells isolated as CD1c^+^, CD14^+^ cells expressed high levels of the M-CSF-receptor CD115, whereas CD14^-^ cells expressed Flt3 (Fig 1C). These data therefore clearly demonstrate that the isolated CD1c^+^ population comprises both cDCs and monocytes. In addition to CD115, CD14^+^CD1c^+^ cells, but not CD14^-^CD1c^+^, expressed further markers considered strongly and specifically expressed by blood monocytes: chemokine receptor CCR2 [25], integrin alpha M (CD11b) [26], triggering receptor expressed on myeloid cells TREM1 [27,28], as well as calprotectin subunit S100A9 [26,29] (Fig 1C).

**Fig 2:**
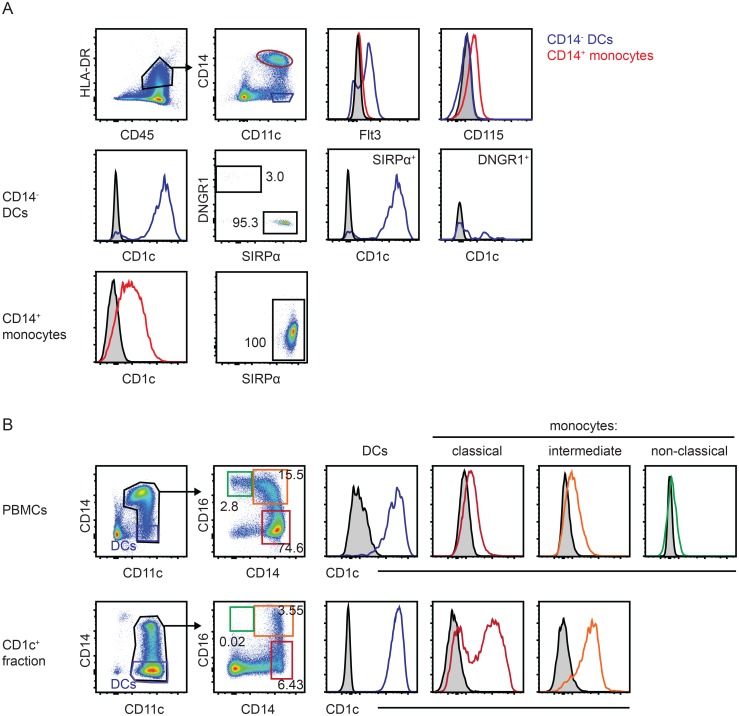
Human classical and intermediate blood monocytes express CD1c. (A) Flow cytometric analysis of CD1c expression among Flt3^+^SIRPα^+^DNGR1^-^ DCs and CD115^+^ monocytes in fresh human PBMCs. Cells are pre-gated on single, live CD45^+^HLA-DR^+^ cells (see S1 Fig). Data are representative for 3 donors. (B) Distribution of human blood monocyte subsets characterized by differential expression of CD14 and CD16 and analysis of their CD1c expression levels before (upper panel) and after (lower panel) application of the CD1c^+^ DC isolation kit. Cells are pre-gated on single, live cells (see S1 Fig).

To further confirm that blood monocytes express CD1c, we verified that among mononuclear phagocytes, CD11c^+^CD14^-^ cells represent Flt3^+^ DCs and CD11c^+^CD14^hi^ cells represent CD115^+^ monocytes (Fig 2A, upper panel). In accordance with the literature [4], among the CD14^-^ DCs, the SIRPα^+^ cDC2 subset expressed high levels of CD1c, while DNGR1^+^SIRPα^-^ cDC1 cells were CD1c^-^ (Fig 2A, center panel). CD115^+^ monocytes were uniformly SIRPα^+^ and largely expressed CD1c, but at levels lower than DCs (Fig 2A, lower panel). In order to determine whether CD1c expression is confined to a specific monocyte subset, we identified the three major blood monocyte populations based on their differential expression levels of CD14 and CD16. CD14^++^CD16^−^ classical and CD14^++^CD16^+^ intermediate monocytes expressed CD1c (Fig 2B, upper panel) and were enriched in the CD1c^+^ population compared with CD1c^-^CD14^+^CD16^++^ non-classical monocytes after bead-based isolation (Fig 2B, lower panel). Among CD1c^+^ cells, DCs and CD14^++^CD16^+^ intermediate monocytes showed a homogeneous expression pattern for CD1c, while CD14^++^CD16^−^ classical monocytes consisted of CD1c^low^ and CD1c^high^ cells (Fig 2B).

**Fig 3:**
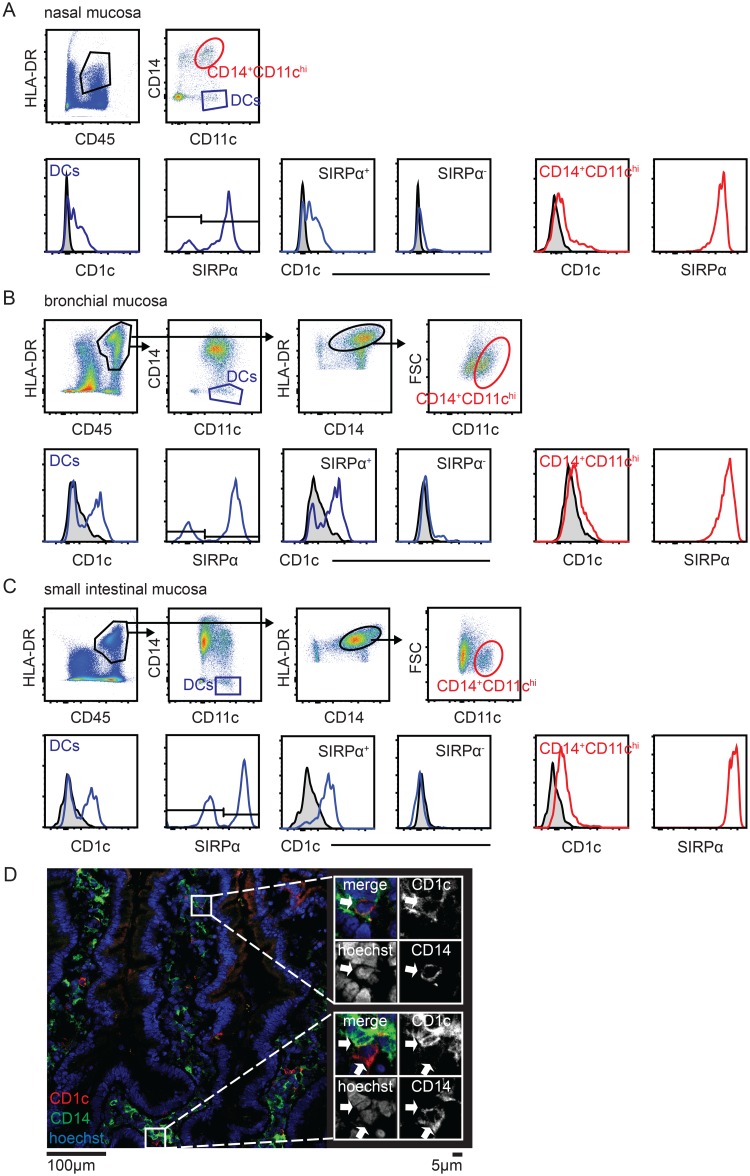
Human mucosal tissue monocytes express CD1c. Flow cytometric analysis of CD1c expression in DCs and monocytes isolated from human (A) nasal mucosa, (B) bronchial mucosa and (C) small intestinal mucosa. Cells are pre-gated on single, live cells. Data are representative for 11, 5 and 59 donors respectively. (D) Expression of CD14 (green) and CD1c (red) was assessed by immunohistochemistry on cryosections from human small intestinal mucosa. Nuclei were stained with hoechst (blue). Images are representative for 4 cryosections each from 3 donors.

Given the expression of CD1c by monocytes in blood, we next investigated whether also monocyte-derived cells in tissue express CD1c. We applied established gating strategies for tissue CD14^-^CD11c^hi^ DCs, and CD14^+^CD11c^hi^ mononuclear phagocytes representing monocyte-derived cells [5, 30, 31] (and Richter et al., Bujko et al., unpublished) on single cell suspensions from healthy mucosal tissues. We found that monocytes in human nasal mucosa (Fig 3A), bronchial mucosa (Fig 3B) as well as small intestinal lamina propria (Fig 3C) express CD1c, albeit at lower levels compared with SIRPα^+^ tissue DCs. Tissue macrophages (CD14^+^CD11c^low^) did not express CD1c at detectable levels (data not shown). We verified this finding by immunofluorescence staining for CD1c and CD14 in cryosections of duodenal/jejunal mucosa. In addition to CD14^-^ DCs expressing CD1c and an abundant population of CD1c^-^CD14^+^ monocytes and macrophages (Fig 3D, lower panel), we identified scattered CD1c^+^CD14^+^ cells in the lamina propria (Fig 3D, upper panel).

### Isolated CD1c^+^ DCs and CD1c^+^ monocytes exhibit different functional properties

As our data demonstrated that bead-sorted CD1c^+^ cells derived from blood are in fact heterogeneous, we sought to investigate potential functional differences between the isolated CD1c^+^ DCs and CD1c^+^ monocytes, thereby determining the impact of the contaminating CD1c^+^ monocytes on assays employed to study CD1c^+^ DC function. To this end, we studied phenotype and function of these cells under diverse culture conditions, upon LPS stimulation and in co-culture with naïve T cells.

**Fig 4:**
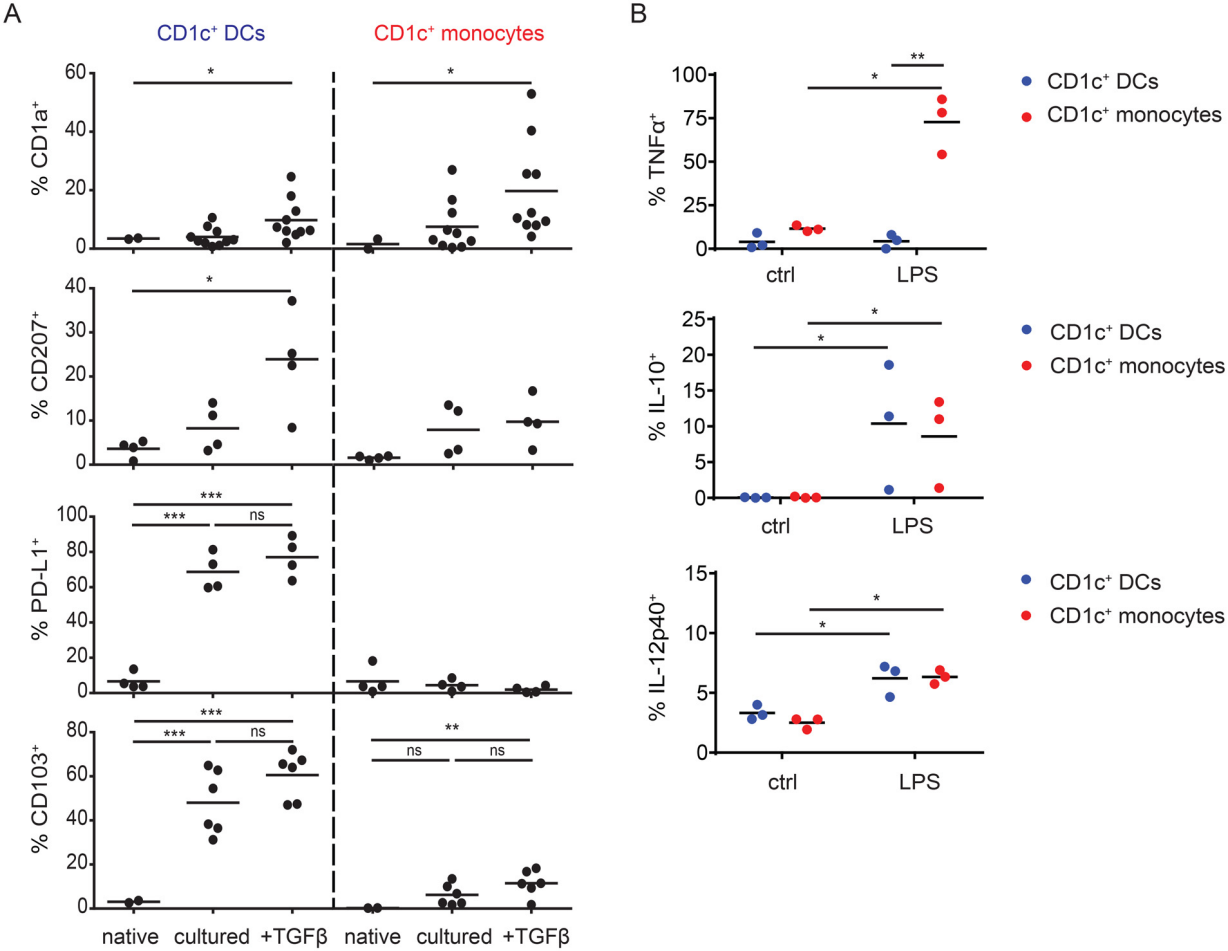
CD1c^+^ DCs and CD1c^+^ monocytes exhibit different properties during culture. (A) Flow cytometric analysis of expression of selected surface markers in CD1c^+^ DCs and CD1c^+^ monocytes in native blood, as well as of CD1c^+^ isolated cells after culture ± 40ng/ml TGFβ for 48h. Data from 2–10 donors. * *P* < 0.05, ** *P* < 0.01, *** *P* < 0.001, **** *P* < 0.0001 (one-way RM-*ANOVA*). (B) Flow cytometric analysis of intracellular TNFα, IL-10 and IL-12p40 in CD1c^+^ DCs and CD1c^+^ monocytes isolated from blood upon stimulation with 1μg/ml LPS for 4.5h, 18h or 24h, respectively. Data from 3 donors. * *P* < 0.05, ** *P* < 0.01 (two-way RM-*ANOVA*).

Expression of CD1a [6,19,32–34] and CD207 [19,35] is induced on blood-derived mononuclear phagocytes upon serum, GM-CSF and/or TGFβ-treatment and expressed on CD1c^+^ DCs in tissues. We found that CD1a was up-regulated upon *in vitro* culture with addition of TGFβ, in both CD1c^+^ DCs and CD1c^+^ monocytes isolated from blood, while only DCs increased CD207 expression after TGFβ-treatment (Fig 4A). Tissue CD1c^+^ cDC2 also express PD-L1 [6,36] and CD103 [4,37,38], with CD103 reportedly induced by TGFβ-signaling [37–39]. PD-L1 was strongly induced in blood CD1c^+^ DCs upon culture, whilst blood CD1c^+^ monocytes consistently remained PD-L1^-^. Blood CD1c^+^ DCs also exhibited a strong up-regulation of CD103 independent of TGFβ, while CD1c^+^ monocytes only displayed marginal increase of CD103 upon TGFβ treatment (Fig 4A). This differential regulation of surface marker expression emphasizes the different properties of CD1c^+^DCs and CD1c^+^ monocytes derived from blood.

Next, we aimed to assess the response to TLR activation in blood-derived CD1c^+^ cells. Upon LPS stimulation, CD1c^+^ monocytes exhibited a significantly greater induction of TNF expression as compared with CD1c^+^ DCs, whereas levels of IL-10 and IL-12p40 production were increased after stimulation with LPS but comparable in both populations (Fig 4B).

**Fig 5:**
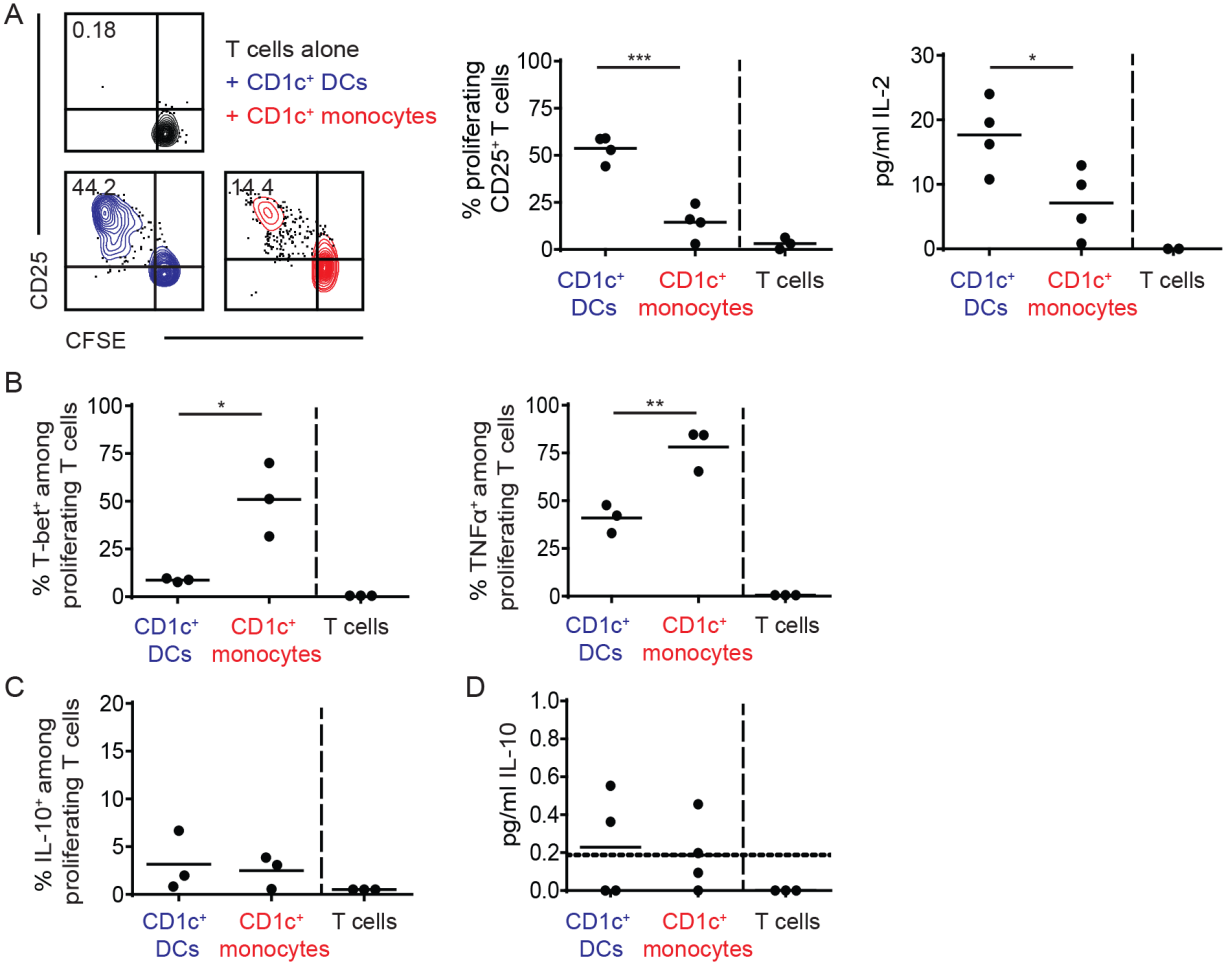
CD1c^+^ monocytes are inefficient at promoting naïve T cell proliferation compared with CD1c^+^ DCs. (A) CFSE-stained naïve CD4^+^ T cells isolated from human blood were cultured for 6d together with CD1c^+^ DCs or CD1c^+^ monocytes and proliferation and expression of CD25 were analyzed using flow cytometry. Cells are pre-gated on single, live CD3^+^ cells. IL-2 levels in the supernatant were assessed using Bio-Plex immune assay after 6 days of coculture. Data are derived from 4 experiments with different mononuclear phagocyte donors. * *P* < 0.05, *** *P* < 0.001 (t-test). (B) T-bet, TNFα and (C) IL-10 expression was assessed among proliferating CD25^+^ T cells after PMA/ionomycin stimulation (3 donors). * *P* < 0.05, ** *P* < 0.01 (t-test) (D) IL-10 concentrations in the supernatant were assessed using Bio-Plex immune assay after 6 days of coculture (4 donors). The horizontal dashed line represents the lowest concentration of the standard curve, data points below were extrapolated.

Finally, in a mixed lymphocyte reaction, CD1c^+^ DCs induced a significantly stronger proliferation of naïve CD4^+^ T cells and higher levels of IL-2 secretion compared with CD1c^+^ monocytes (Fig 5A). However, among the T cells that were stimulated by monocytes, a significantly higher fraction produced TNF after PMA/ionomcyin stimulation and expressed T-bet as compared with T cells primed by DCs (Fig 5B). This indicates a stronger skewing towards a proinflammatory Th1 response by CD1c^+^ monocytes. Neither DCs nor monocytes induced considerable numbers of IL-10-producing T cells (Fig 5C and 5D).

### Monocyte pre-depletion increases of purity of CD1c^+^ DCs

**Fig 6:**
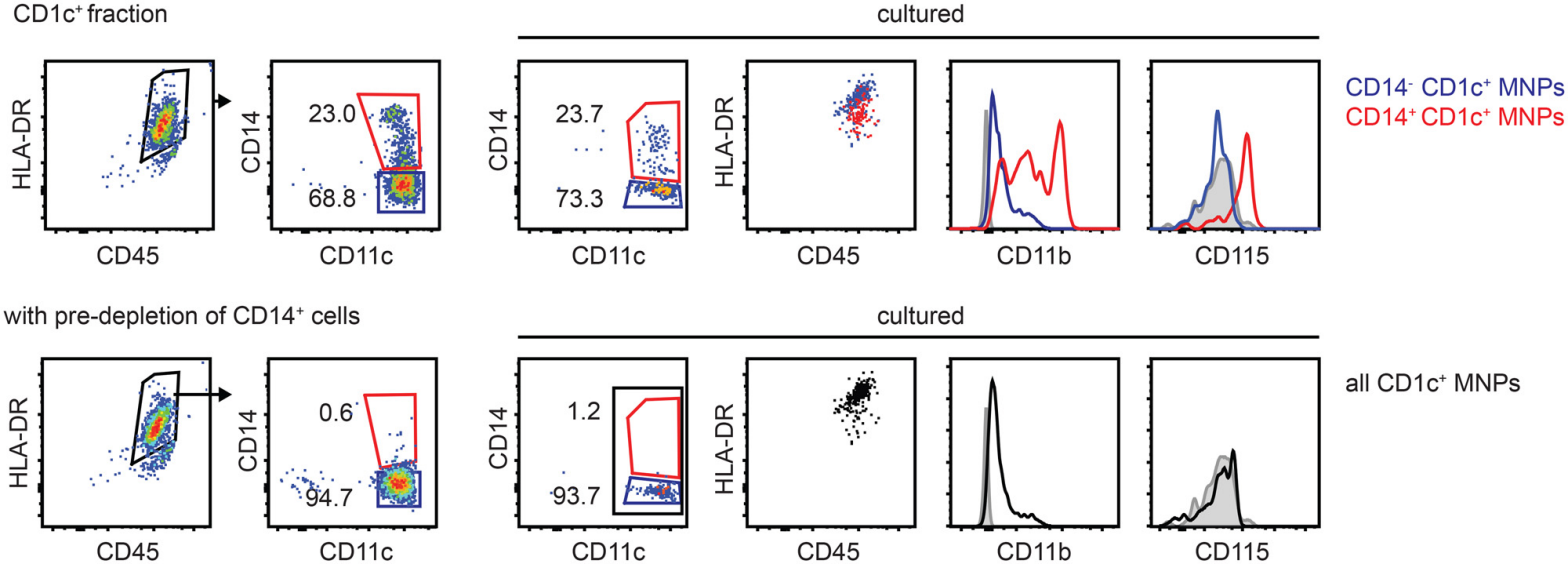
Pre-depletion of monocytes increases purity of DCs in isolated CD1c^+^ population. Flow cytometric phenotyping of the resulting CD1c^+^CD14^-^ and CD1c^+^CD14^+^ populations after application of the CD1c^+^ DC isolation kit without (upper panel) and with (lower panel) pre-depletion of CD14-expressing cells using CD14 MACS micro beads according to the manufacturer’s instructions. Cells are pre-gated on single, live cells. Data are representative for 3 donors.

Given the different functional properties of CD14^+^CD1c^+^ and CD14^-^CD1c^+^ cells we aimed to provide a protocol for further purification of the subsets which is feasible without FACS-sorting. We exploited the expression of CD14 by monocytes for their depletion from PBMCs, followed by application of the CD1c (BDCA-1)^+^ Dendritic Cell Isolation Kit. This pre-depletion of CD14^+^ cells lead to high enrichment of CD14^-^CD11b^low^ DCs. in the CD1c^+^ population with homogenous phenotype after culture and virtually complete absence of CD14^+^CD11b^high^CD115^+^ monocytes (Fig 6).

## Discussion

We here demonstrate that a subset of monocytes expresses CD1c, and that a widely used commercial CD1c (BDCA-1)^+^ Dendritic Cell Isolation Kit isolates both CD1c^+^ DCs and CD1c^+^ monocytes. We furthermore show that these two populations have strikingly different properties, complicating the interpretation of phenotypical and functional properties of CD1c^+^ DCs isolated using this protocol.

While early publications describing CD1c as a cDC marker had used lin^-^ cell populations for the characterization of CD1c^+^ DCs [3], CD1c has since often been used as sole marker for cDC2 cells among mononuclear phagocytes. However, early work also suggested certain heterogeneity within the cell population isolated solely based on CD1c, with dichotomous expression patterns for markers such as CD14, HLA-DR, CD83 and CD86 induced in culture [1]. We here resolve this heterogeneity by demonstrating that the CD1c^+^ population consists of CD14^-^ DCs and CD14^+^ monocytes with clear differences in phenotypical and functional properties.

CD1c^+^ monocytes only promoted low level proliferation of CD4^+^ T cells compared with CD1c^+^ DCs, in line with reports that monocytes and monocyte-derived tissue cells have a low capacity to prime naïve T cells [4,40–43]. Murine infection models indicate that monocyte-derived cells are important for the initiation of Th1 immunity [44–46], and indeed the proliferating T cells induced by CD1c^+^ monocytes were primarily T-bet^+^ Th1 cells.

In our hands, the percentage of CD14^+^ monocytes among the bead-isolated CD1c^+^ mononuclear phagocyte population showed great inter-individual variation, ranging from 25 to 70%. Accordingly, treating the CD1c^+^ mononuclear phagocyte population as one entity will result in bias of the generated data depending on the donor-specific relative abundance of CD14^+^CD1c^+^ mononuclear phagocytes. Our results indicate that data-sets describing the expression of cell surface markers after culture [1,13,14], cytokine secretion and responsiveness to stimulation [11–15] as well as T cell activation [13–15] might be influenced due to contamination of a presumed CD1c^+^ cDC2 population by monocytes. Also, without further distinction between CD14^+^ and CD14^-^CD1c^+^ mononuclear phagocytes, phenotypical and functional comparisons of the presumed CD1c^+^ cDCs with other cell subsets such as plasmacytoid DCs or monocytes [12,17] might be inaccurate. With every research group working with CD1c^+^ cells investigating different combinations of stimuli and read-outs, our data cannot provide a complete insight into the differential properties of CD14^+^CD1c^+^ and CD14^-^CD1c^+^, but rather highlight that the potential effect of contamination with CD14^+^ should be assessed for each experimental setting.

While we used FACS to isolate CD14^++^ and CD14^-^ cells in the CD1c^+^ fraction resulting from the CD1c (BDCA-1)^+^ Dendritic Cell Isolation Kit for our functional studies, laboratories that want to study CD1c^+^ cDC2 cells but do not have access to FACS-based sorting can reduce the contamination of the population by pre-depleting cells expressing typical monocyte surface markers which are not expressed by the majority of cDC2, as we have shown here for CD14.

Collectively, our findings demonstrate that CD1c is expressed by a subset of monocytes and contribute to a further dissection of the heterogeneity within the mononuclear phagocyte system. In addition, our results suggest that studies on CD1c^+^ DCs isolated using CD1c-targeting alone need to be interpreted with caution.

## Supporting Information

S1 Fig Gating strategy and characterization of cell populations. (A) Gating strategy for flow cytometry analysis of antigen-presenting cell populations in PBMCs before and after application of the CD1c (BDCA-1)^+^ Dendritic Cell Isolation Kit. Arrows indicate sequential gating. Data are representative for 6 donors. (B) Characterization of cell populations in the isolated CD1c^+^ fraction versus the cell fractions depleted during the application of the kit (combined CD19^+^ cells and CD1c^-^ cells). Arrows indicate sequential gating. Data are representative for 4 donors. (C) Quantification of the cell populations as determined in (B). Data are given as mean±SD from 4 donors.
(TIF)
