# Supplementary figures and images for: CD1c-Expression by Monocytes – Implications for the Use of Commercial CD1c+ Dendritic Cell Isolation Kits
Source: PLoS One. 2016 Jun 16;11(6):e0157387. doi: 10.1371/journal.pone.0157387 (PMC4911075; doi:10.1371/journal.pone.0157387)

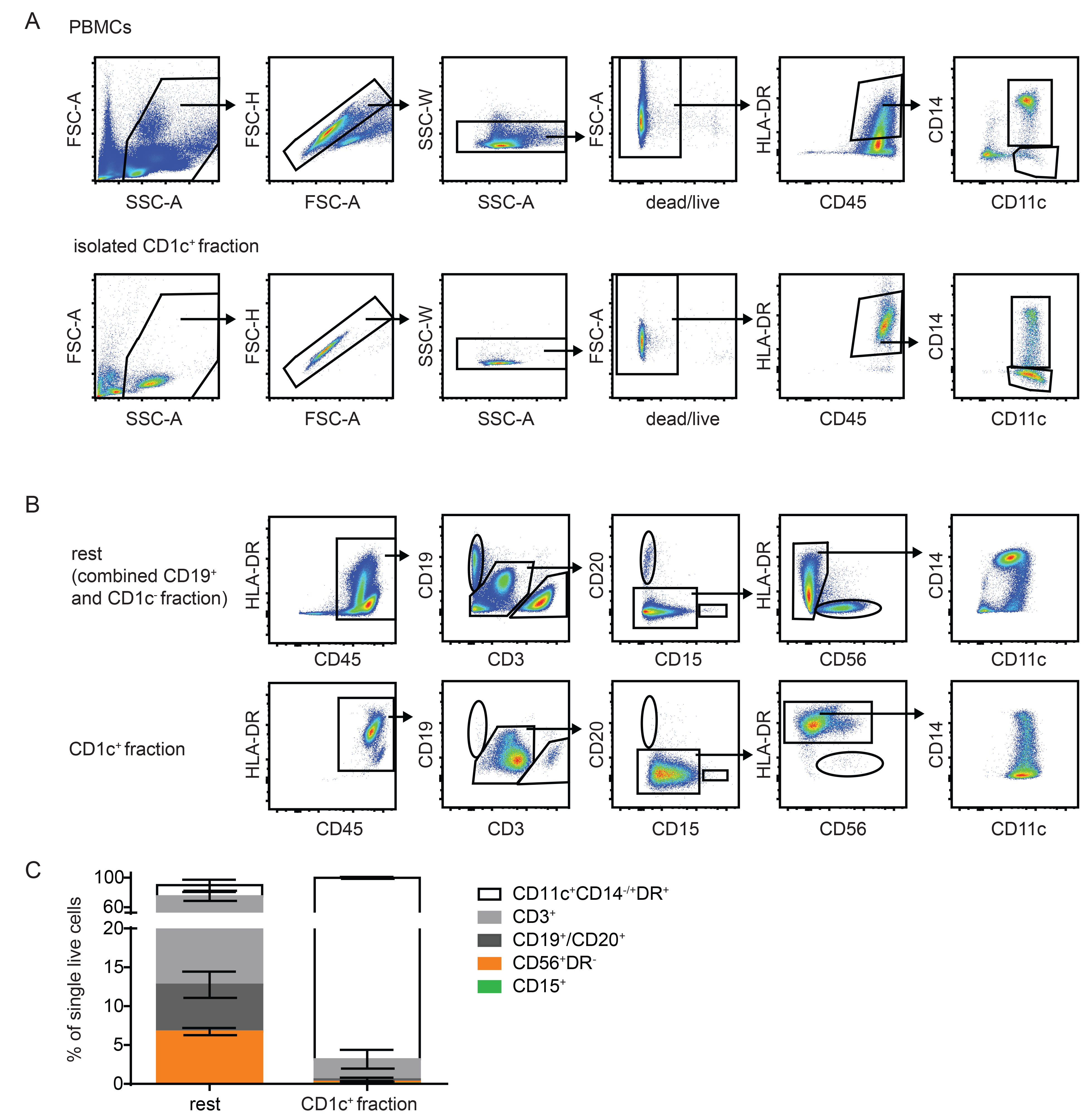

Supplement: S1 Fig — (A) Gating strategy for flow cytometry analysis of antigen-presenting cell populations in PBMCs before and after application of the CD1c (BDCA-1)+ Dendritic Cell Isolation Kit. Arrows indicate sequential gating. Data are representative for 6 donors. (B) Characterization of cell populations in the isolated CD1c+ fraction versus the cell fractions depleted during the application of the kit (combined CD19+ cells and CD1c- cells). Arrows indicate sequential gating. Data are representative for 4 donors. (C) Quantification of the cell populations as determined in (B). Data are given as mean±SD from 4 donors. (TIF) [file pone.0157387.s001.tif]
